# Hot electrons in water: injection and ponderomotive acceleration by means of plasmonic nanoelectrodes

**DOI:** 10.1038/lsa.2017.2

**Published:** 2017-06-30

**Authors:** Pierfrancesco Zilio, Michele Dipalo, Francesco Tantussi, Gabriele C Messina, Francesco de Angelis

**Affiliations:** Istituto Italiano di Tecnologia, 16163, Genova, Italy

**Keywords:** hot electrons, hydrated electrons, lightwave electronics, plasmonics, ponderomotive acceleration, strong-field photoemission, water breakdown

## Abstract

We present a theoretical and experimental study of a plasmonic nanoelectrode architecture that is able to inject bunches of hot electrons into an aqueous environment. In this approach, electrons are accelerated in water by ponderomotive forces up to energies capable of exciting or ionizing water molecules. This ability is enabled by the nanoelectrode structure (extruding out of a metal baseplate), which allows for the production of an intense plasmonic hot spot at the apex of the structure while maintaining the electrical connection to a virtually unlimited charge reservoir. The electron injection is experimentally monitored by recording the current transmitted through the water medium, whereas the electron acceleration is confirmed by observation of the bubble generation for a laser power exceeding a proper threshold. An understanding of the complex physics involved is obtained via a numerical approach that explicitly models the electromagnetic hot spot generation, electron-by-electron injection via multiphoton absorption, acceleration by ponderomotive forces and electron-water interaction through random elastic and inelastic scattering. The model predicts a critical electron density for bubble nucleation that nicely matches the experimental findings and reveals that the efficiency of energy transfer from the plasmonic hot spot to the free electron cloud is much more efficient (17 times higher) in water than in a vacuum. Because of their high kinetic energy and large reduction potential, these proposed wet hot electrons may provide new opportunities in photocatalysis, electrochemical processes and hot-electron driven chemistry.

## Introduction

The possibility to generate free electrons in water has been attracting interest for several decades in many different fields of chemical and physical sciences because of their extremely high reactivity^1^. Indeed, free electrons can be considered the most powerful and simple reducing agents in chemistry, showing huge reduction potentials, exceeding more than −5 eV with respect to the normal hydrogen electrode^1^. Free electrons have a fundamental role in many photochemical or electrochemical processes, and they participate as a trigger or an intermediate state in an extremely wide variety of chemical, biological or physical processes. However, although the intense studies have been dedicated to them since the 1970s^2^, many aspects remain unclear. The difficulties originate from the wide range of energy and time scales involved in these processes. For example, the time landscape can span from femto- to micro-seconds, thus making computation methods such as molecular dynamics less effective. Most of the current knowledge comes from experiments of the radiolysis of water produced by a high-energy electron beam or intense laser radiation, which usually result in very different and complex outcomes.

Recently, electron driven processes gained further attention because of their favorable combination with plasmonic nanostructures^3, 4, 5^, opening the path to plasmon driven photo-electrocatalytic processes^6, 7^. The latter appears to be greatly promising because it can combine the capability of plasmonic nanostructures of harvesting optical energy and producing the so-called hot electrons^6, 7, 8, 9, 10^, namely energetic electrons that are not in thermal equilibrium with their environment. As a result, plasmonic hot spots are emerging as an ideal tool for triggering electro-photochemical reactions that otherwise present very low efficiencies^6^. The injection of hot electrons into vacuum^11, 12, 13^, solid^9, 14, 15, 16^ or liquid^17, 18, 19, 20, 21^ media is being extensively investigated for many applications. However, we notice that, whereas the physics of the injection in solid devices is largely understood^9^, the physical and chemical behavior of hot electrons in liquids is far more complex^17, 18, 19, 20, 21^ and still presents many issues that must be clarified for this field to evolve. Towards this goal, it would be extremely useful to have a controllable and effective source of free electrons in water, more precisely; electrons that are not transferred to molecules adsorbed onto the metal surface but are directly injected into the water environment. Furthermore, it would be important to have the capability of increasing the kinetic energy of injected electrons above the thresholds for water excitation (4–6 eV) and water ionization (10–12 eV)^1^.

In this work, we report experimental and theoretical results of hot-electron injection in water, and the ponderomotive acceleration of the electrons by means of plasmonic nanoelectrodes illuminated by femtosecond laser pulses. Namely, a plasmonic hot spot is exploited to enhance multiphoton absorption from a nanotip-like electrode that causes electron injection from metal to water. We show that once free electrons are injected in water, they can be accelerated by the plasmonic field through the ponderomotive process^13^. Under proper conditions, free electron kinetic energy can exceed tens of eV, that is, above the threshold for water excitation, ionization and secondary electron avalanche generation. As will be described below, we implemented a comprehensive multiphysics model adopting an electron-by-electron simulation approach that yields good agreement with the experimental data and provides clear and visual insight regarding the whole process. Importantly, we show that the elastic collisions of free electrons with water molecules makes the free electron cloud more confined, thus enhancing the ponderomotive energy transfer. Therefore, the latter results tend to be more effective in water than that occurring in a vacuum (a factor 17 of enhancement).

**Figure 1:**
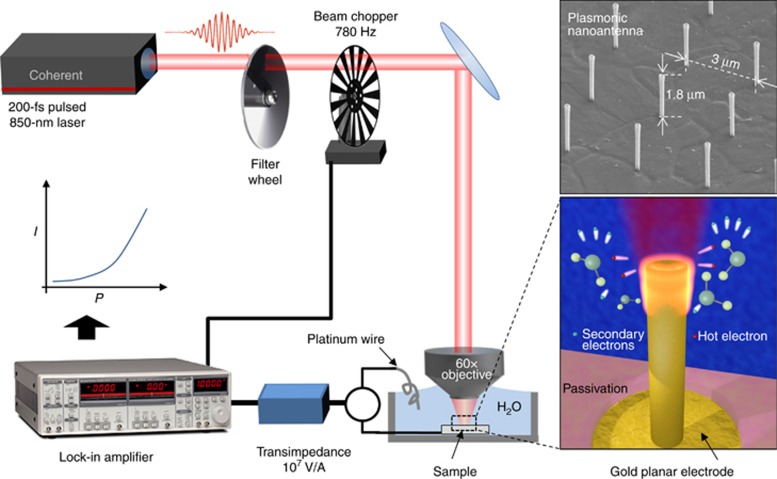
Electro-optical setup for measuring the electron current produced by femtosecond laser excitation. The inset reports a scanning electron microscope image of a fabricated antenna.

The experimental setup is sketched in Figure 1. As shown in the figure, we exploited a 3D vertical plasmonic nanoantenna protruding from planar electrodes^22, 23, 24^. This configuration shows the following different important advantages: (i) the injection of electrons in water can be verified and quantitatively measured by following the electrical current flow through the electrodes; (ii) heat generated at the antennas tip is dissipated into the electrode and substrate without damaging the antennas, as often occurs when nanoparticles are used in similar experiments^18^; and (iii) the electrode acts as a metal reservoir, thus compensating the charging effects via hot-electron emissions^25^. In such a manner, there is no decrease in the efficiency of the system, in contrast to plasmonic nanoparticles, where charge carrier recombination should be counterbalanced to preserve the efficiency of the emission phenomenon^26^. In other words, free electrons can be steadily generated at each optical cycle and then accelerated without being affected by the restoring force that typically rules the exciton dynamics.

Importantly, the optical setup enables the observation of cavitation bubbles for laser powers higher than a certain threshold and demonstrates the effectiveness of this electron acceleration, as described below.

## Materials and methods

Gold planar electrodes were evaporated on quartz samples and then connected to external pads by means of gold tracks. Arrays of gold vertical nanotubes (1800-nm-tall, 90-nm outer radius, 60-nm inner radius, 3-μm pitch) were fabricated by means of secondary electron lithography^22, 27^, a technique based on focused ion beam milling of a silicon nitride membrane coated by an S1813 resist layer. Gold tracks finally bring the contact outside the sample. In this way, it is possible to measure the electron current at the nanoantenna/water interface during laser excitation. To electrically insulate the conductive tracks from the deionized water, a 2-μm SU-8 photoresist passivation layer was deposited on the whole sample. This passivation layer was patterned by optical lithography to expose the planar electrodes with the nanoantennas to the water. A scanning electron microscope image of an antenna array is shown in the inset of Figure 1, which also shows a schematic of the electro-optical measurement setup.

In the experiment, a tunable laser (Coherent MIRA900 with Coherent Verdi G10 pump, Coherent Inc., Santa Clara, CA, USA) was used as the light source, for which the wavelength was tuned in the near infrared at 850 nm, and the emission was in pulses with 200-fs pulse width at 76-MHz repetition rate. The pulsed beam is then chopped at 780 Hz with a chopper wheel and fed to an upright WiTec microscope. The laser is focused onto the nanoantenna tip by means of a 60× immersion objective (NA=1) that produces a laser spot with a beam waist of ~700 nm, as estimated experimentally by a Gaussian fit of the intensity profile. The sample with the nanoantennas is immersed in MilliQ grade deionized water and is electrically connected to a transimpedance with 10^7^ V/A gain (Femto DHPCA-100, FEMTO Messtechnik GmbH, Berlin, Germany). A platinum wire immersed in the deionized water acts as counter-electrode for the current measurements; all measurements of photocurrent are made without the application of a bias between the platinum counter-electrode and the sample with nanoantennas. The transimpedance output is fed to the input of a lock-in amplifier (Stanford Research Systems SR830, Stanford Research Systems, Inc., Sunnyvale, CA, USA), which locks the signal to the chopper frequency. The SU-8 passivation on the sample ensures that there are no leakage currents between the platinum wire and the other gold surfaces on the sample.

A rotating gradual filter wheel was used to change the laser intensity while the current at the nanoantenna/water interface was measured. By means of a photodiode connected to the same oscilloscope where the output of the lock-in amplifier was connected, we were able to simultaneously measure the laser intensity and the relative generated current. For each laser intensity setting, we measured the generated current while the excitation spot was moved between nanoantennas and the planar gold substrate to allow the corresponding measured currents to be compared.

To simulate the complex physics involved in our system, we developed a model implemented in the COMSOL Multiphysics simulation environment. Its description in full detail is reported in the Supplementary Information.

**Figure 2:**
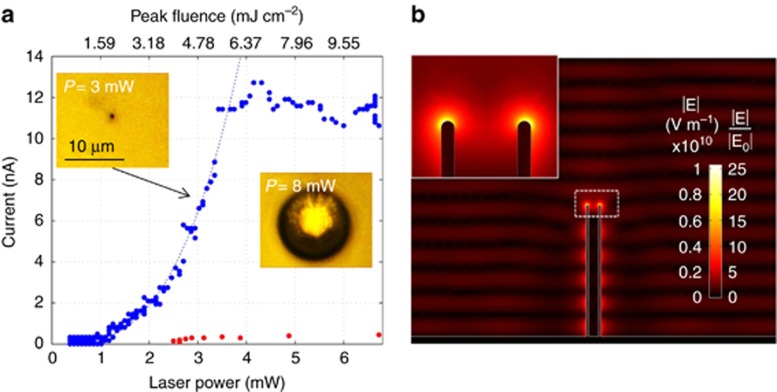
(**a**) Measured photocurrent as a function of impinging laser power in the presence of nanoantenna (blue circles) and in the case of bare gold coated membrane (red circles). The dotted lines mark the fit with the power law *i*(*t*)=*AP*^3^. Insets show optical images of one antenna illuminated by light with power below and above the breakdown threshold (*P*=3 mW and 8 mW, respectively), the latter showing the presence of a cavitation bubble. (**b**) Simulated electromagnetic field norm distribution around the plasmonic antenna. The two-color scales report, respectively, the values in the case of *P*=5 mW (left scale) at the pulse peak and the values normalized to the maximum impinging field amplitude at focus (right scale). Inset: detail of the field at the tip.

The electromagnetic field distribution around the nanoantenna (an example is reported in Figure 2b) is obtained by solution of the time harmonic Helmholtz equation, assuming a normally impinging linearly polarized plane wave with unitary amplitude. The time-dependent electromagnetic field produced by the focused pulsed beam, *E*(*x*,*t*), is then obtained by properly renormalizing the field to the space and time maximum of the pulse and by multiplying by the temporal pulse shape provided by the laser datasheet (in our case a sech^2^-like time dependence). In particular, the range of laser powers considered is 1–6 mW. All other parameters of the model are fixed to match the experimental ones.

The electron photoinjection in water is modeled by adopting the experimentally found photocurrent functional behavior with respect to the impinging power, *I*=AP^3^ (see also the ‘Results and Discussion’ section and ‘Discussion’ therein). The input to the charged particle tracing simulation is the photoemission current density 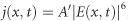, where the constant *A′* is obtained from the fit constant *A* upon proper renormalization (Supplementary Information). This procedure avoids the explicit modeling of the photoemission process, which critically depends on the local work function of the gold–water interface and, in turn, depends on the gold roughness and the space-dependent temperature distribution. The correct space and time dependence of the current density is given by the electric field enhancement distribution. Electrons are injected according to *j*(*x*, *t*) with an initial kinetic energy equal to 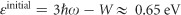.

With respect to the current literature, here, we do not use macroscopic plasma-fluidodynamics equations to model the effect of the free electrons in water. Instead, we consider a more fundamental level, explicitly modeling the electron-by-electron injection and dynamics in the water environment in the presence of the plasmonic hot spot. The electron trajectories are obtained by solution of the equation of motion subject to the time-dependent force produced by the plasmonic electromagnetic field and to stochastic deviations produced by collisions with water molecules. Based on a very recent paper in the literature^28^, we consider the details of the integral and differential cross section in the angular deviation for the elastic collisions by interpolation/extrapolation of the reported experimental data. For inelastic collisions, we take into account the ionization and excitation differential inverse mean free paths, calculated from evaluation of the electron loss function, as has been described extensively in the references^29, 30, 31, 32, 33^.

We observe that the model considers the water properties at a molecular level as follows: the ionization pathways for the five molecular orbitals (1*a*_1_, 2*a*_1_, 1*b*_2_, 3*a*_1_, 1*b*_1_) of the H_2_O molecule in the liquid phase, five excitation levels (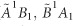, Ryd A+B, Ryd C+D, diffuse bands), exchange effects and semi empirical low-energy corrections to improve the reliability of the model at low energies^29^. Recombination is neglected in the model because it occurs in times much longer than the pulse duration^21^.

We highlight the importance of the developed model because the overall number of electrons produced by primary and secondary emission is not very large at the considered impinging light intensities, ranging from few tens to some hundreds of thousands of electrons. Therefore, at the lowest powers, it is expected that macroscopic transport equations do not provide a realistic picture. However, the limited electron number allows an explicit electron track simulation to be conducted at reasonable times.

## Results and discussion

Figure 2a reports the measured currents (*i*) read from the electrode as a function of the laser power (*P*) impinging onto the sample. The measured photocurrent is the result of the charge transfer between the gold antenna and water when hot electrons are ejected from the former and transferred to the latter during laser excitation. We compare the typical current read from the electrode by on-axis illumination of one antenna (blue circles) with the one obtained from flat gold. The antenna yields a current larger by a factor 40, for *P*>3.5 mW. Clearly, this is related to the plasmonic hot spot produced at the antenna termination, as shown in Figure 2b, where we report a finite element simulation of the electric field norm around the antenna. The left color scale reported refers to the case *P*=5 mW at the peak of the pulse, and the right scale shows the enhancement with respect to the impinging beam field at focus. Two different regimes are clearly found within the spanned power range. Below a certain threshold of *P*=P*≈3.5 mW, the current follows a power law dependence on the input power, reaching maximum values of ~12 nA. From a fit of the data with a functional form of *i*(*t*)=*AP*^*n*^, we obtain *A*=0.231±0.029 nA/(mW)^*n*^ and *n*=3.023±0.11. Such a dependence suggests that the main emission mechanism in this power range can be identified as 3-photon absorption^32^. This absorption is expected because the impinging photons at *λ*=850 nm have an energy of 1.46 eV, that is, the simultaneous absorption of three photons is required to exceed the gold–water work function, whose value is *W*=3.72 eV^34^.

Above P*, the current shows an irregular oscillatory behavior around the saturation value of 11 nA. A visual inspection by optical microscopy reveals the formation of a cavitation bubble for powers *P*>8 mW (Figure 2a, inset). However, the refresh time of our camera is relatively long, 160 ms. According to the recent literature, the cavitation bubble dynamics at threshold conditions is much faster (~100 ns); thus, we expect the actual threshold for cavitation to be at *P*<8 mW. On the other hand, the sudden departure from the power law behavior observed at P*=3.5 mW suggests that this is the threshold for nanobubble formation. Indeed, the bubble partially screens the nanoelectrode ends and changes the local refractive index, producing both a decrease in the number of photons reaching the antenna apex and a reduction of the expected field enhancement^17, 18, 19, 20, 21, 35^. These effects likely compensate the increase of the impinging light power, thus determining the observed saturation in the *i*–*P* curve.

The calculated peak fluence corresponding to the threshold power of *P*=3.5 mW is ~5.5 mJ cm^−2^. As a comparison, for an 800-nm wavelength, 200-fs-long pulse, the fluence required for optical breakdown in pure water has been reported to be ~800 mJ cm^−2^ (Refs 18, 21), while that yielding a relevant plasma-related bubble formation in off-resonant gold nanoparticles is ~200 mJ cm^−2^ (Refs 17, 18). The fluence causing bubble formation in a resonant gold nanoparticle has been reported to be much lower, ~9 mJ cm^−2^; however, in this case, the effect has been related to the huge energy absorption and consequent temperature increase within the gold nanoparticle, leading to damage or fragmentation of the particle itself^17, 35^. This effect can be reasonably excluded in the present case because of the efficient heat dissipation provided by the gold baseplate. Indeed, no alteration of the structures or the current response was observed in the considered power range (for more details, see the Supplementary Information). Nano- and micro-bubble generation by resonant gold nanoparticles has been investigated in the literature in the case of continuous-wave laser excitation^36^. Here, the plasmonic electric fields due to a continuous-wave laser are orders of magnitudes lower and cannot extract electrons from the gold–water interfaces; bubble formation is shown to be produced by the large temperature increase originating from energy absorption.

When plasmonic nanostructures are excited with laser pulses in the visible/near infrared range in the femto-/pico-second regime, they can emit electrons in free space by means of photoelectric emission. Such a process has been extensively studied in vacuum for the generation of highly energetic electron bunches^11, 12, 13^. Explicit modeling of the photoemitted electrons from illuminated plasmonic sources has been presented by Dombi *et al.*^11, 12, 13^ In those papers, the authors propose a model consisting of the following three separate steps: electromagnetic absorption into the plasmonic structure, electron injection by multiphoton or field emission and ponderomotive acceleration by the plasmon-enhanced electromagnetic field. According to that well-established model, once the electron is emitted into the vacuum, its energy is equal to its initial kinetic energy plus its potential energy arising from the fact that it is immersed in an electric field potential. Because the electron is free to move, the potential energy will be converted into kinetic energy. That process is usually called ponderomotive acceleration and has been largely investigated in vacuum conditions. In this work, we invoke the same description to explain the free electron dynamics in the water environment, showing that the presence of elastic collisions makes the process much more effective.

Let’s now consider the generation of free electrons in water, which is usually achieved by using strongly focused femtosecond pulse without the use of plasmonic structures. A widely used schematic description^21^ considers water on the femtosecond landscape to behave as an amorphous semiconductor with a band gap Δ=6.5 eV^37^. Once a free electron is produced in water by laser ionization (excited to the conduction band according to the formalism of the field of semiconductors), it can gain kinetic energy through a process called inverse bremsstrahlung. If the electromagnetic field is strong enough, then the electron gains sufficient energy to produce secondary electrons through impact ionization and, above a proper laser power threshold, it may result in avalanche generation and plasma formation. Under these conditions, a strong energy transfer from the plasma to the water produces a vapor bubble. In particular, Vogel *et al.*^21^ determined that, for *λ*=800 nm, a density ρ*≈0.236 nm^−3^ defines the cavitation threshold in pure water. The free electron density is, therefore, a crucial parameter. More recently, it has been shown that the insertion of noble metal nanoparticles within the focus of a high-energy laser pulse in water leads to strong reduction in the threshold laser fluence for bubble nucleation^17, 18, 19, 20, 38^.

This phenomenon has been explained by considering the intense electromagnetic field enhancement surrounding the metal nanoparticles, which, depending on the light intensity, may either induce the water ionization and subsequent generation of a highly absorptive plasma^17, 18, 19, 20^ or simply determine a strong light absorption into the nanoparticle, with consequent heat transfer to the water environment^38^.

**Figure 3:**
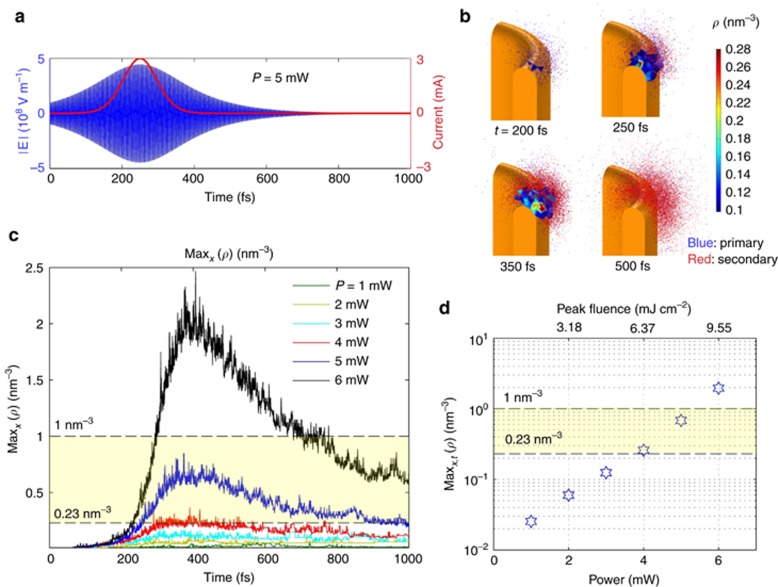
(**a**) Impinging pulse electric field at the focus (blue) for *P*=5 mW and instantaneous photoemitted current (red). (**b**) Snapshots of the calculated electron clouds for *P*=5 mW taken at four time instants. Primary and secondary electrons are colored with blue and red, respectively. Superimposed are volume plots enclosing the regions where the free electron density exceeds 0.1 nm^−3^. (**c**) Calculated spatial maximum of the free electron density around the gold nanoantenna for impinging power ranging from 1 to 6 mW. (**d**) Space- and time-maxima of the free electron density as a function of the laser power. In **c** and **d** the yellow band marks the range in which the threshold density is expected, according to the literature^21^.

In Figure 3, we report the results of the simulation of 200-fs pulses, for different values of the focused light power. The time dependence of the impinging electric field at the tip apex is reported in Figure 3a in the case of *P*=5 mW together with the corresponding instantaneous current assumed in the simulation, according to the experimental fit. The simulation allows for the direct study of the evolution of the electron cloud that develops around the antenna. In Figure 3b, we report four snapshots at *t*=200, 250, 350 and 500 fs for *P*=5 mW. Primary and secondary electrons are colored in blue and red, respectively.

As mentioned above, the crucial parameter to be monitored during the simulation is the free electron density, *ρ*. We calculate the free electron density by counting the number of electrons within the local mesh elements. In Figure 3b the volume plots superimposed to the electron cloud plots encloses the mesh elements where *ρ* exceeds 0.1 nm^−3^. The maximum above-threshold electron-density is found to be close to the plasmonic hot spot on the metal surface and is confined to a distance of a few nm.

Figure 3c reports the time evolution of the spatial maximum of *ρ* for impinging powers ranging from 1 to 6 mW. The figure shows that *ρ* rapidly fluctuates with the time-varying electric field and strongly increases with the impinging power in a non-linear way. Figure 3d reports the time maxima of the curves reported in Figure 3c as a function of *P*. According to the calculations, the maximum density exceeds the critical density in literature of 0.23 nm^−3^ that is required for breakdown at *P*=4 mW, and further exponentially increases to almost 10 times this value at *P*=6 mW. The predicted threshold value excellently matches the experimental result. A minor quantitative mismatch can be reasonably attributed to charge accumulation effects that grow pulse after pulse and likely occur at the considered pulse repetition rates (76 MHz)^21^ but are neglected in the simulation.

**Figure 4:**
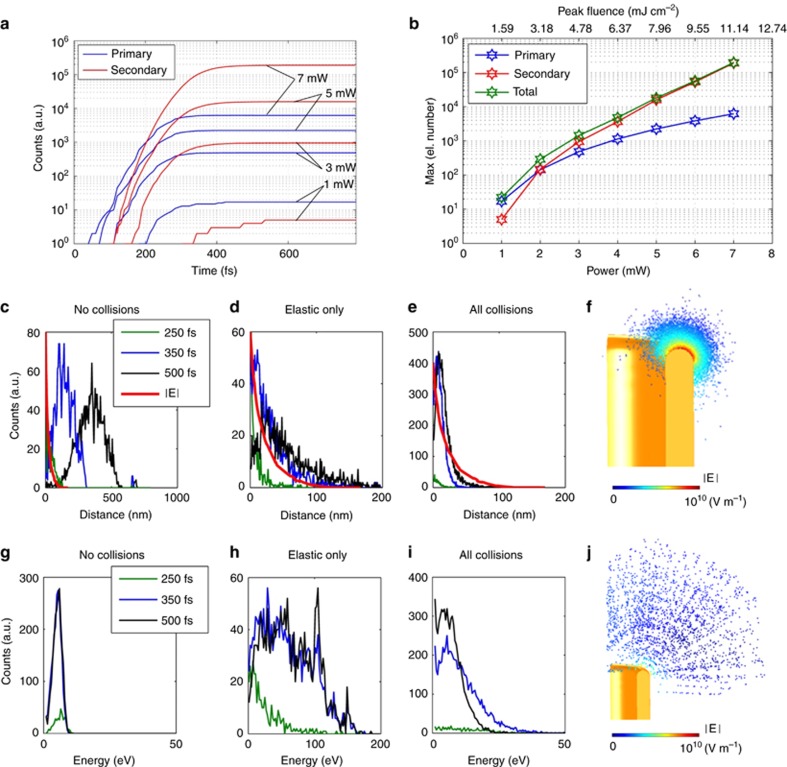
(**a**) Calculated primary (blue) and secondary (red) electron numbers as a function of time for impinging laser powers from 1 to 7 mW. (**b**) Maximum primary secondary and total electron numbers as a function of laser power. (**c**–**e**) Plots of the electron distance distributions from the gold surface for three time instants, in the case of the absence of collision (**c**), elastic collisions only (**d**) and both elastic and inelastic collisions (**e**); (**c**–**e**) plots share the same legend. The red line represents the normalized electric field distribution close to the hot spot (**f**) plot of the electron cloud at *t*=500 fs, the color scale represents the electric field norm at the electron position. (**g**–**i**) Electron energies distribution in the same cases as in **c**–**e**. (**j**) Same plot as in **f**, but in the absence of collisions. All of the plots **c**–**j** are calculated for *P*=5 mW.

In Figure 4a, we show the number of primary and secondary electrons generated by the pulse as a function of time, while Figure 4b reports the electron numbers at the last simulation time versus power. These plots reveal that the growth of the secondary electrons is much faster than that of the primary, namely, than the growth of the net current. From the plots, at the pulse end, the secondary electrons number exceed the primary ones for *P*>2 mW and rapidly become the large majority of the total number of free electrons in water with increasing power. From the log-plot, it is clear that the growth is approximately exponential for *P* higher than 3.5 mW. This result is consistent with the experimental observation of a very clear power threshold in the *i*–*P* curve of Figure 2a. At this power, we count 790 emitted primary electrons per pulse, while the secondary electrons exceed 2000 in number.

We remark that the ionization number explosion is entirely produced by the plasmonic hot spot, which at the same time determines an enhanced photoemission from the metal surface and enables the free electron acceleration up to energies high enough to ionize the water molecules. Importantly, we found that a key role is played by the elastic scattering in water, which prevents the electron cloud from moving far away from the metal surface and keeps it close to the hot spot. This process is shown in Figure 4c–4j, where we compare the distributions of electrons distances from the gold surface (Figure 4c–4e) and electron energies (Figure 4g–4i) in the absence of collisions (Figure 4c and 4g), in the presence of elastic collisions only (Figure 4d and 4h) and including both elastic and inelastic collisions (Figure 4e and 4i). The distributions are shown in the case of *P*=5 mW for three time instants, *t*=250 (green), 350 (blue) and 500 fs (black). The red line reports a normalized plot of the plasmonic electric field norm at the hot spot as a function of the distance from the metal surface. As shown in the figure, in the absence of collisions (this is the case for vacuum environment^13^) the electrons gain kinetic energy only during the first optical cycles, quickly moving away from the hot spot location and reaching a maximum kinetic energy of just 10 eV. The presence of elastic collisions dramatically changes the electron spatial and energetic distributions, keeping the electron cloud extremely close to the hot spot with a consequent strong and repeated acceleration of electrons. We notice that the effectiveness of the acceleration depends on both the strong field and the strong-field gradient produced by the hot spot. In fact, in the presence of a strong but uniform oscillating field, electrons experience a zero average acceleration. The inclusion of ionization events in the simulation results in an even stronger confinement of the electron cloud within a maximum distance of 50 nm at *t*=500 fs. This is consistent with the literature data of electron penetration range^1^. The overall energy transfer to the water for *P*=5 mW turns out to be 35 fJ, whereas in case of absence of collisions, we obtain 2 fJ, namely a factor 17 lower. Thus, despite its extreme localization, the plasmonic field enhancement is efficiently exploited for the energy transfer to the electrons for most of the pulse duration, with the result being that the critical laser fluence required for breakdown is two orders of magnitude lower than that one required to produce it in pure water (5.5 mJ cm^−2^ and 800 mJ cm^−2^, respectively, as reported above)^21^. Moreover, it is a very important point that the energy transfer from plasmonic field to free electrons is much more effective when the process occurs in water (or liquid) rather than in vacuum.

Note that, unlike the case of nanoparticles floating in water, in the proposed structure, electron photoemission from the metal-water interface has a significant role. In fact, at the threshold power *P*=3.5 mW for example, the experimental current of 12 nA corresponds to the injection of ~1100 electrons per pulse, which spread at an average distance of 30 nm from the metal surface, as shown in Figure 4c. As can be easily calculated, if an analogous number of electrons were photoemitted by a gold nanoparticle, an electric field of the order of 10^8^ V m^−1^ or higher would arise between the electron cloud and the positively charged particle (assuming roughly a particle radius of 100 nm and a uniform particle-cloud distance of 30 nm). The actual value is likely to be higher because of the strong localization of the electron emission at the particle poles, where the field is higher. These field values are comparable to the impinging light field (Figure 3c), thus canceling out the plasmonic field enhancement responsible for the electron injection.

We remark that the interest of our study is not related to bubble generation, which was used just to prove the existence of ponderomotive acceleration. We also remark that here the primary electrons are ejected and accelerated by the plasmonic field and must be distinguished from hydrated electrons or solvated electrons, which refer to electrons that are slowing down (hydrated) or have already thermalized (solvated) with the water environment, that is, trapped in a cavity formed by water molecules^1^.

The proposed plasmonic architecture turns out to be a localized, efficient and well-controlled source of accelerated free electrons that are not in thermal equilibrium with water molecules. In analogy with hot electrons in solid media, they could be defined as wet hot electrons. Interestingly, such electrons are rapidly separated from the emitting electrode. In fact, these electrons can travel across water environment, where they can react with the solute without being affected by the metal surface. This simplified configuration may help to clarify those reaction mechanisms in which the participation of the electrode material is undesired. Furthermore, the spatial separation between the hot carriers and the emitting electrode prevents both hot carrier recombination and electrode degradation. Being extremely reactive because of their kinetic energy and huge reduction potential, these electrons can be very useful for triggering and investigating many different chemical and physical processes currently poorly understood or otherwise extremely inefficient. These hot electrons can give important contributions in many different fields in which free electrons have a major role, such as photocatalysis and electrochemical process^39^, hot-electron driven chemistry^6^, water radiolysis^40^, hydrogen generation^41^ (including that coming from nuclear waste)^40^, fundamental studies on hydrated electrons and solvated electrons^1^, hyperthermia with gold nanoparticles and plasmonic photothermal therapy^42^, DNA damaging^1^ and others not reported here for brevity.

## Conclusions

In this work, we presented experimental data and a numerical model that describe hot-electron injection and acceleration in water by photoexcitation of 3D plasmonic nanoelectrodes. The injection was experimentally monitored by measuring the electric current flowing into the water, whereas the ponderomotive acceleration of the electrons was confirmed by the observation of cavitation bubbles.

We implemented a multiphysics model that considers the electromagnetic field distribution around the antenna, the electron photoemission, the ponderomotive acceleration of electrons and their interaction with water molecules through elastic scattering, inelastic scattering and secondary electron generation by means of ionization events. The model results were found to be in very good agreement with the experimental data, and the model can be useful for further investigations of electron injection in liquids, leading to more efficient generation and exploitation of hot electrons in various fields of chemistry, physics and biology. Interestingly, the model directly reveals how the elastic scattering helps to maintain the electron cloud overlapped to the plasmonic hot spot, thus determining a much more efficient (a factor 17 higher) energy transfer to the electrons than in the case of emission in vacuum. Moreover, the use of 3D plasmonic antennas connected to flat electrodes offers an infinite reservoir of electrons that would allow the long-term and steady generation of wet hot electrons. The latter result may be of great importance when continuous-wave illumination or sunlight is used to trigger electron injection, thus opening a path toward energy production without the requirement of catalysts or reducing agents.
