# Supplementary material for: Hot electrons in water: injection and ponderomotive acceleration by means of plasmonic nanoelectrodes
Source: Light Sci Appl. 2017 Jun 30;6(6):e17002–. doi: 10.1038/lsa.2017.2 (PMC6062236; doi:10.1038/lsa.2017.2)
Supplement: Supplementary Information [file lsa20172x1.docx]

Supplementary Information for

Hot electrons in water: injection and ponderomotive acceleration by means of plasmonic nanoelectrodes

Pierfrancesco Zilio^1‡^, Michele Dipalo^1‡^, Francesco Tantussi^1^, Gabriele C Messina^1^ and Francesco de Angelis^1^

^1^ Istituto Italiano di Tecnologia, via Morego 30, 16163, Genova Italy

**1. Model description**

The model we adopt for the simulation of light pulse absorption, electron emission, acceleration and interaction with water molecules follows the approach of the semiclassical simple-man model,^1,2^ in which the main physical processes are decoupled in three steps of simulation:

1. Electromagnetic simulation of the optical response of our plasmonic nanoelectrode;
2. Space and time dependent electron emission;
3. Solution of electron equation of motion subject to the plasmonic field acceleration and interaction with water molecules.

All of the steps are performed by using COMSOL Multiphysics simulation environment. In what follows we describe in detail these steps. We also describe in some details some COMSOL implementation issues, in order to facilitate the reader interested in reproducing the model.

1. **Electromagnetic simulation**

The electromagnetic field distribution has been calculated by solution of the Helmholtz equation, by means of the finite elements method. The simulation setup is shown in Fig. S1a. The simulated plasmonic structure consists of a hollow vertical gold antenna standing on a gold substrate, embedded in water dielectric environment. Material refractive indexes for gold and water are taken from literature.^3^ Fixed geometrical parameters are considered throughout the paper, namely height of 1800 nm, outer radius of 90 nm, gold thickness of 30 nm. The curvature radius of the top edge is assumed to be 15 nm, according to the measured values from scanning electron microscopy (See inset of Figure 1 in the main text). A plane wave illumination is set with normal incidence and polarization along the x axis. Perfect Electric Conductor (PEC) and Perfect Magnetic Conductor (PMC) boundary conditions are set on the y-z and x-z planes respectively. Perfectly Matched Layers are displaced all around the antenna in order to absorb scattered and back reflected light.


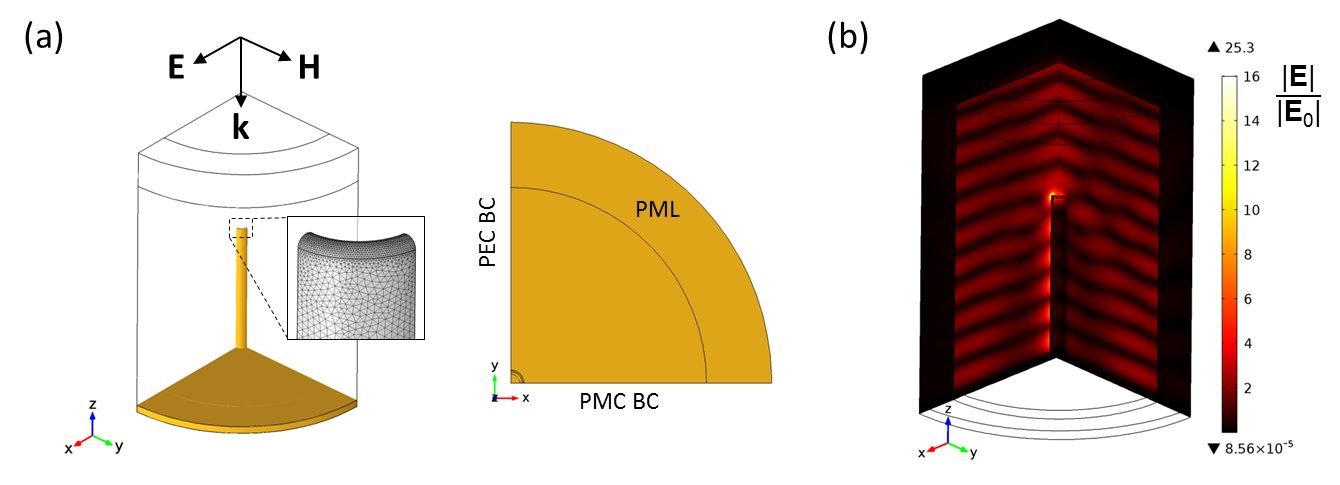


Figure S1. (**a**) 3D scheme and top view of the electromagnetic simulation; (**b**) Electric field norm distribution in x-z and y-z cross sections.

The simulation enables to calculate the field enhancement distribution around the antenna (Fig. S1b), which we call hereafter *f*(**x**). In the experiment the illumination consists of a strongly focused pulsed Gaussian beam (objective numerical aperture NA = 1, pulse duration *τ_p_* = 200 fs, repetition rate r = 76 MHz, pulse shape, according to the vendor data sheet, I(t) = p(t)^2^ = {sech[(t-t_0_)∙1.76/*τ_p_*]}^2^), with power ranging from 1 to 10 mW. The focus z-coordinate is set on the tip end. In the simulation, an approximation of the real field distribution at the tip apex is obtained by $E(x,t)=E_{0}f(x)p(t)e^{i\omega t}\hat{e}_{n}$ being $\hat{e}_{n}$ the local unit vector normal to the metal surface and *E*_0_ the peak electric field on focus. This is obtained as $E_{0}=\sqrt{2I_{0}Z_{0}/n}$, where *Z*_0_ is the vacuum permittivity, *n* = 1.33 is the water refractive index and *I*_0_ is the peak intensity at the focus center, $I_{0}=2\cdot0.88\cdot E_{p}\cdot(\tau_{p}\pi w_{0}^{2})^{-1}$, where w_0_ is the beam waist, *E_p_* is the pulse energy and the factor 0.88 takes into account the sech2 shape of the pulse.^4^ The beam waist *w*_0_ has been experimentally estimated by Gaussian fit of the intensity profile at focus. The obtained value is *w*_0_ ≈ 700 nm.

It is important to notice that the maximum impinging beam peak intensity at focus, for a laser power of 10 mW, is about 70 GW cm^-2^, which is nearly two orders of magnitude lower than the typical ones required to produce the water breakdown.^5^ The detail of the spatial field distribution of the impinging beam is therefore irrelevant in our case, since in absence of any plasmonic structure no substantial electron injection from water molecules is expected at the focus position. It is therefore meaningful to simulate the focused beam as a plane wave, which strongly reduces the computational time.

1. **Electron injection model**

In the second step of the model, the space and time dependent electron photocurrent is calculated from the field enhancement distribution.

As shown in the main text, experimental measurements show that the functional dependence of the current on the impinging power *P* is of the kind $i(P)=AP^{3}$, where *A* = 0.2319 nA mW^-3^, being compatible with a 3-photon absorption mechanism. We therefore set in the model this experimental current dependence, by imposing the total emitted current to be *i*(*P*) and by assuming for the space and time dependent emission current density the following equation:

$j(x,t)=A'\left|E(x,t)\right|^{6}$ (S1)

The constant *A’* is obtained by imposing

$i(P)=AP^{3}=r\int_{0}^{1/r} \int_{\Sigma} A'\left|E(x,t)\right|^{6}dtd\sigma$ (S2)

where Σ is the antenna surface area and *r* is the repetition rate. Explicitly,

$A'=A\cdot r^{2}\left(\frac{4\cdot0.88\cdot Z_{0}}{\pi w_{0}^{2}\tau_{p}n}\right)^{-3}\left(\int_{0}^{1/r} p(t)^{6}\right)^{-1}\left(\int_{\Sigma} f(x)^{6}\right)^{-1}.$ (S3)

In the model, electrons are emitted with spatial distribution proportional to *j*(**x**,t) with velocity vector normal to the local metal surface orientation and with initial kinetic energy given by , being *W* the gold-water work function, 3.72 eV.^6^

1. **Calculation of the electron trajectories**

In the third and final step, the electrons classical equation of motion is solved subject to the local oscillating electric field (due to both the impinging light wave and the plasmonic field enhancement) and to interactions with water molecules. We use the COMSOL particle tracing module for the calculation. A total simulation time of 1 ps is assumed, while the solver time step is automatically set by the software in order to resolve the trajectories evolutions. A “bounce” boundary condition is set at the emitting gold surface, with this meaning that electrons driven back to the metal surface are specularly reflected.^2,7^

Three main electron-water interactions are taken into account: elastic scattering, excitations, ionizations.^8^

For the elastic differential and total scattering cross sections as a function of the electron energy and scattering angle we consider an interpolation of recent literature data provided by Matsui et al.,^9^ where an electron energy range from 2 to 100 eV is covered, well fit to the present expected electron energy range. The scattering direction is determined from the differential cross section by means of the inverse transform method, namely inverting the cumulative distribution function.^10^

For ionization and excitation inelastic collisions we follow the theory presented by Dingfelder^11,12^ and Emfietzoglou.^8,13–15^ Within the frame of the first Born approximation, the probability of energy transfer *E* and momentum transfer *ħE* is proportional to the energy loss function (ELF), given by $\mathrm{Im}[-1/\varepsilon(E,q)]$, where $\varepsilon(E,q)$ is the water dielectric response function, ^8,13–15^ namely

$\frac{d^{2}\Sigma}{dEdq}(T,E,q)=\frac{1}{\pi\alpha_{0}Tq}\mathrm{Im}\left[\frac{-1}{\varepsilon(E,q)}\right].$ (S4)

In this expression *Σ* is the *macroscopic cross section*, in units of (length)^-1^, or the *inverse mean free path*, *α_0_* is the Bohr radius, *T* is the kinetic energy of the incident electron, *E* and *q* are respectively the transferred energy and momentum. The energy loss function for water at *q* = 0, is directly obtained from optical data measured by Heller et al.^16^ The extension to all kinematically possible values of *E* and *q*, the so-called Bethe surface of the material^12^ is the achieved following the Fully Extended Drude (FED) model developed by Emfietzoglou et al.^14^. In this model the experimental ELF for *q* = 0 is analytically represented as a sum of “normal” and “derivative” forms of the Drude-type functions. For the sake of completeness, we report here the final formula and the set of parameters we considered with their meanings (Table S1). We remand to the cited references for further details.

$\mathrm{Im}\left[\varepsilon(E,q=0)\right]_{\exp}=\left\{\begin{matrix} \sum_{j}^{ioniz} D_{2}(E,f_{j},E_{j},\gamma_{j})+\sum_{j}^{excit} \tilde{D}_{2}(E,f_{j},E_{j},\gamma_{j}), for E>B_{ioniz,\min} \\ \sum_{j}^{excit} \tilde{D}_{2}(E,f_{j},E_{j},\gamma_{j}) for B_{excit,\min}\leq E<B_{ioniz,\min} \end{matrix}\right.$ (S5)

$\mathrm{Re}\left[\varepsilon(E,q=0)\right]_{\exp}=1+\sum_{j}^{ioniz} D_{1}(E,f_{j},E_{j},\gamma_{j})+\sum_{j}^{excit} \tilde{D}_{1}(E,f_{j},E_{j},\gamma_{j})$ (S6)

$D_{1}(E,f_{j},E_{j},\gamma_{j})=E_{p}^{2}\frac{f_{j}\left(E_{j}^{2}-E^{2}\right)}{\left(E_{j}^{2}-E^{2}\right)^{2}+\left(\gamma_{j}E\right)^{2}}$ (S7)

$\tilde{D}_{1}(E,f_{j},E_{j},\gamma_{j})=E_{p}^{2}\frac{f_{j}\left(E_{j}^{2}-E^{2}\right)\left[\left(E_{j}^{2}-E^{2}\right)^{2}+3\left(\gamma_{j}E\right)^{2}\right]}{\left[\left(E_{j}^{2}-E^{2}\right)^{2}+\left(\gamma_{j}E\right)^{2}\right]^{2}}$ (S8)

$D_{2}(E,f_{j},E_{j},\gamma_{j})=E_{p}^{2}\frac{f_{j}\gamma_{j}E}{\left(E_{j}^{2}-E^{2}\right)^{2}+\left(\gamma_{j}E\right)^{2}}$ (S9)

$\tilde{D}_{2}(E,f_{j},E_{j},\gamma_{j})=E_{p}^{2}\frac{2f_{j}\gamma_{j}^{3}E^{3}}{\left[\left(E_{j}^{2}-E^{2}\right)^{2}+\left(\gamma_{j}E\right)^{2}\right]^{2}}$ (S10)

The momentum dependence is introduced empirically extending the parameters *f_j_, E_j_, γ_j_* to the momentum space, based on analyses of experimental data,^14^

$f_{excit,j}(q)=f_{excit,j}\left[\exp(-a_{j}q^{2})+b_{j}q^{2}\exp(-c_{j}q^{2})\right]$ (S11)

$f_{ioniz,j}(q)=f_{ioniz,j}\left(1-\sum_{j}^{excit} f_{excit,j}(q)\right)\cdot\left(1-\sum_{j}^{excit} f_{excit,j}\right)^{-1}$ (S12)

$E_{ioniz,j}(q)=E_{ioniz,j}+\frac{q^{2}}{2m_{e}}\left[1-\exp(-cq^{d})\right]$ (S13)

$\gamma_{j}(q)=\gamma_{j}+aq+bq^{2}$ (S14)

Dingfelder’s energy-loss function^11,12,17^ comprises the ionization pathways for the five molecular orbitals (1*a*_1_, 2*a*_1_, 1*b*_2_, 3*a*_1_, 1*b*_1_) of the H_2_O molecule in the liquid phase. The model includes exchange effects and low-energy corrections to improve reliability at energies below few hundreds of eV. five excitation levels ($\tilde{A}^{1}B_{1},$ $\tilde{B}^{1}A_{1},$Ryd A + B, Ryd C + D, diffuse bands^11^). The differential cross section for excitation (superscript “k”) and ionization (superscript “j”) are then obtained from the dielectric response function as

$\frac{d\Sigma^{exc}}{dE}(E,T)=\sum_{k}^{exc} \Phi_{k}(T)\frac{d\Sigma^{k}}{dE}(E,T)$ (S15)

$\frac{d\Sigma^{ion}}{dE}(E,T)=\sum_{j}^{ion} \Phi_{j}(T)\frac{d\tilde{\Sigma}^{j}}{dE}(E,T)$ (S16)

where

$\frac{d\Sigma^{k/j}}{dE}(E,T)=\frac{1}{\pi a_{0}T}\int_{K_{\min}(E,T)}^{K_{\max}(E,T)} \frac{\mathrm{Im}[\varepsilon^{k,j}(E,T)]}{[\mathrm{Re}(\varepsilon(E,T))]^{2}+[\mathrm{Im}(\varepsilon(E,T))]^{2}}\frac{dT}{T}$ (S17)

The energy transfer ranges up to *E*_max_ = *T*, and the momentum transfer from *K*_min_ to *K*_max_, given by

(S18)

(S19)

In equation (16), the ionization differential cross sections $d\Sigma^{j}/dE$ are substituted with the following expression,^11^ which takes into account electron exchange corrections

$\begin{matrix} \frac{d\tilde{\Sigma}^{j}}{dE}(E,T)=\frac{d\Sigma^{j}}{dE}(E,T)+\frac{d\Sigma^{j}}{dE}(T+E_{j}-E,T) \\ -\left(1-\sqrt{\frac{E_{j}}{T}}\right)\left(\frac{d\Sigma^{j}}{dE}(E,T)\cdot\frac{d\Sigma^{j}}{dE}(T+E_{j}-E,T)\right)^{1/2} \end{matrix}$ (S20)

In addition, the empirical functions $\Phi_{k,j}(T)$ were introduced by Dingfelder^11^ in order to extend the model validity to low electron energies,

$\Phi_{k,j}(T)=\left\{\begin{matrix} 0 for T<E_{k,j} \\ 1-\exp[d_{j,k}(T/E_{k,j}-1)] for T>E_{k,j} \end{matrix}\right..$ (S21)

In the following table we summarize the parameter values assumed in the model:

Table S1: Parameter values assumed in the model

| Parameter | Values | Description |
| --- | --- | --- |
| *E_j_* | 11.95, 14.70, 16.60, 33.3, 540 (eV) | Ionization parameters of the dielectric response function. j=1-5 correspond respectively to shells 1*a*_1_, 2*a*_1_, 1*b*_2_, 3*a*_1_, 1*b*_1_^11^ |
| *γ_j_* | 12.5, 16.1, 19.4, 95, 220 (eV) |
| *f_j_* | 0.3400, 0.3100, 0.2394, 0.1595, 0.3110 |
| *B_j_* | 10, 13, 17, 32.2, 539.7 (eV) | Ionization thresholds ^14^ |
| *E_k_* | 8.17, 10.13, 11.31, 12.91, 14.50 (eV) | Excitation parameters of the dielectric response function. k=1-5 correspond respectively to the excited states $\tilde{A}^{1}B_{1},$ $\tilde{B}^{1}A_{1},$Ryd A + B, Ryd C + D, diffuse bands^11^ |
| *γ_k_* | 1.62, 2.2, 2.1, 3.1, 3.9 (eV) |
| *f_k_* | 0.0118, 0.023, 0.01675, 0.0285, 0.028 |
| *B_e_* | 7 (eV) | Excitation threshold^13^ |
| *a_j_* | 3.82, 2.47, 2.47, 3.01, 2.44 | Parameters for the momentum transfer dependence^11,13^ |
| *b_j_* | 0.0272, 0.0295, 0.0311, 0.0111, 0.0633 |
| *c_j_* | 0.098, 0.075, 0.074, 0.765, 0.425 |
| *a, b, c, d* | 10,6,1.5,0.4 |
| *E_p_* | 21.46 (eV) | Plasmon energy for liquid water ^14^ |
| *d_k_* | -0.25, -0.25, -0.25, -0.25, -0.25 | Semi-empirical low energy correction factors ^11^ |
| *d_j_* | -0.14, -0.18, -0.21, -0.40 |

1. **Further details on the COMSOL Implementation**

The calculations were entirely performed within the COMSOL Multiphysics simulation environment (version 5.2).

The electromagnetic part of the simulation makes use of the RF module of COMSOL. A time harmonic scattering analysis is performed, assuming the field of the multilayer stack without any antenna as a background field. This is calculated from Fresnel laws of reflection and refraction.^18,19^

The simulation of the space charge cloud makes use of the Particle Tracing Module of COMSOL. The software allows to easily and automatically solve for the electron trajectories once the electron release and interactions with background water environment are accurately specified.

For what concerns release, we adopted an “Inlet” boundary condition. This release condition was preferred among the other possibilities offered by the program since it allows to consider an arbitrarily shaped emitting surface and to easily set a local release density proportional to an arbitrary surface quantity. In particular, in our case, we considered as the release surface the nanoantenna outer surface, while the local emission density has been set to be proportional to the photoemission current density as calculated by Equation S1.

The time dependence of Equation S1 required some effort to be implemented in COMSOL, since the software (up to version 5.2) does not allow random emission times nor a time-dependent emission number. The emission instants were arbitrarily chosen at regular time intervals (once every three optical cycles). The time dependent number of electron released at each emission instant has been set by adopting the following procedure. First we calculate the maximum number of emitted electrons during the pulse as $N_{\max}=Q_{p}/(e\cdot\int p(t)^{6}dt)$ , being *Q_p_* the total emitted charge per pulse and *p*(*t*) the pulse shape as defined in section 1. This is set as the number of particles per release. In order to modulate the number of emitted particle according the time dependent current density, j(**x**,t), we define the uniformly distributed random variable number$\xi$in the range [-1,0] and let the i-th electron to be emitted with velocity $v=\sqrt{2E_{k,0}/m_{e}}$ if $\xi_{i}+p(t)^{6}>0$ or with velocity $v=\tilde{V}$ otherwise, being $\tilde{V}$ a very large velocity (for example, 10^8^ m s^-1^). Electrons emitted with velocity $\tilde{V}$are then filtered out in the post-processing stage, and do not undergo any scattering event.

For what concerns the electron inelastic collisions, differential scattering cross sections for ionization and excitation as a function of electron energy and energy loss, Equations S15 and S16, have been calculated numerically by means of a code external to the COMSOL environment. In order to randomly sample the energy loss from the respective distributions defined by the differential cross sections, we adopted the inverse-transform method,^20^ namely we numerically evaluated the inverses of the cumulative functions. Then, matrices of values for the energy losses, $\Delta E_{ion}(T,u)$ and $\Delta E_{exc}(T,u)$, as a function of the input electron energy T and of a uniformly distributed random variable *u* in the range [0,1], are imported in COMSOL and interpolated with the built-in program functionalities.

An analogous procedure was followed to randomly sample the doubly differential (energetic and angular) elastic cross section. The angular dependence of the inelastic differential cross section is instead neglected, and random directions are set according to a uniformly distributed random unitary vector.

**2. Mechanical/thermal stability of the structure**

The stability of 3D plasmonic nanoantennas during electron emission has been tested by exciting the same antenna for several seconds with a varying laser power. The laser power is cycled by a motorized rotating filter wheel that span from 0.2 mW to 7 mW roughly every half a second. The instantaneous laser power and the corresponding emission current from the antenna are simultaneously measured by a photodiode and by a transimpedance connected to a lock-in amplifier.

In Fig. S2 the laser power (black line) and the emission current (red line) from the same antenna are plotted versus time. From this graph two important observations can be made. First of all, the instantaneous emission current follows very well the varying exciting power. Secondly, and most importantly, the emission current level among the power cycles is stable and does not show signs of degradation. Considering the laser repetition rate of 76 MHz and the total amount exciting time, we have observed that the antennas are stable for more than 10^8^ laser pulses.

The small current oscillations visible in Fig. S2 can be correlated with mechanical vibrations that change the alignment of the laser spot over the antenna.


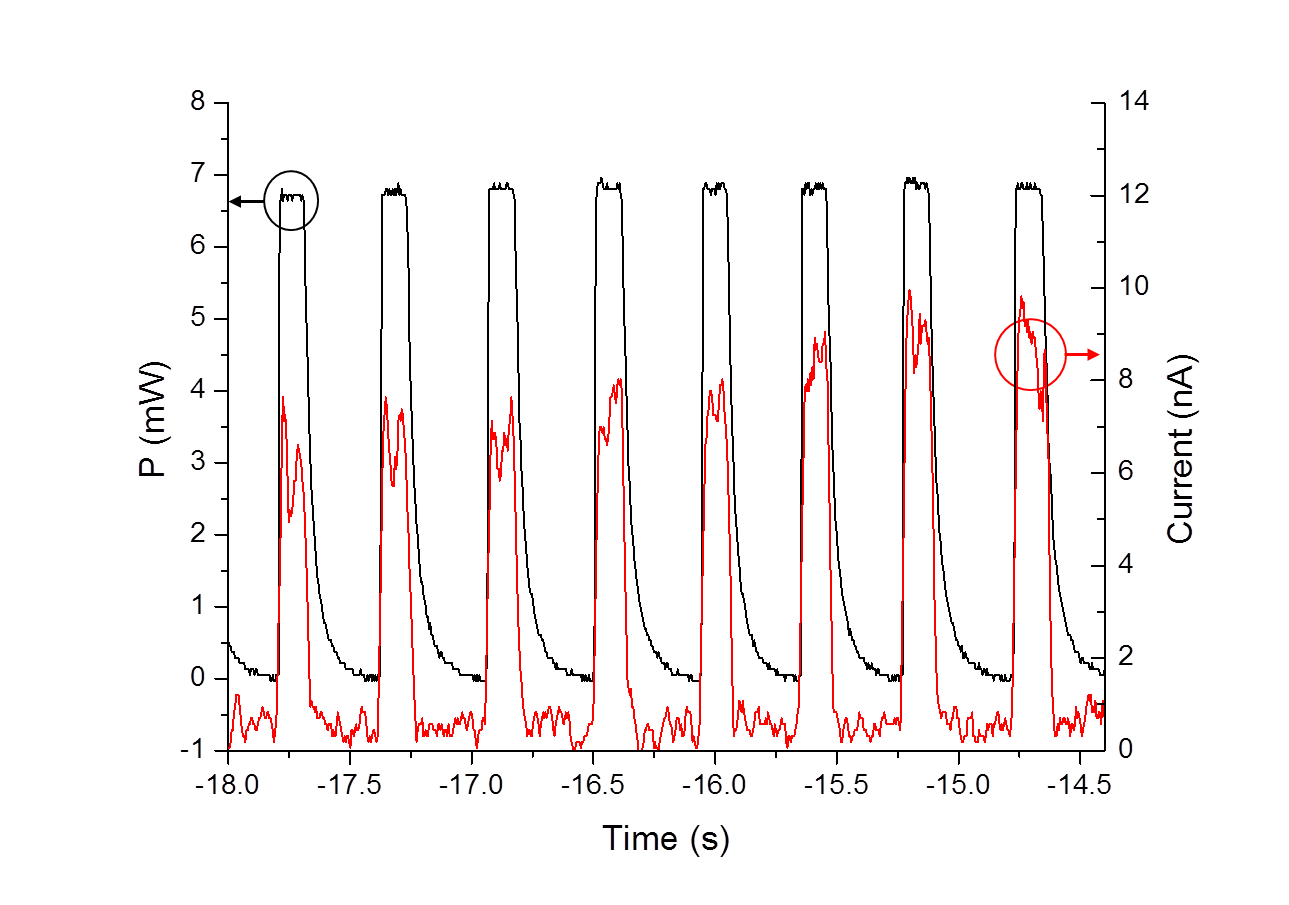


Figure S2: Time evolution of emission current from the same antenna while the exciting laser power is varied.

**3. Absorption spectrum of 3D nanoantenna**

Fig. S3 shows the simulated absorption spectrum of 3D nanoantennas with geometry and size like those used for the experiments. As can be seen in the plot, several plasmonic resonances are present and the height of the antenna had been tuned in order to match a resonance at the working wavelength (850 nm). The validity of the model used for this simulation has been proved experimentally in previous works.^21,22^

**
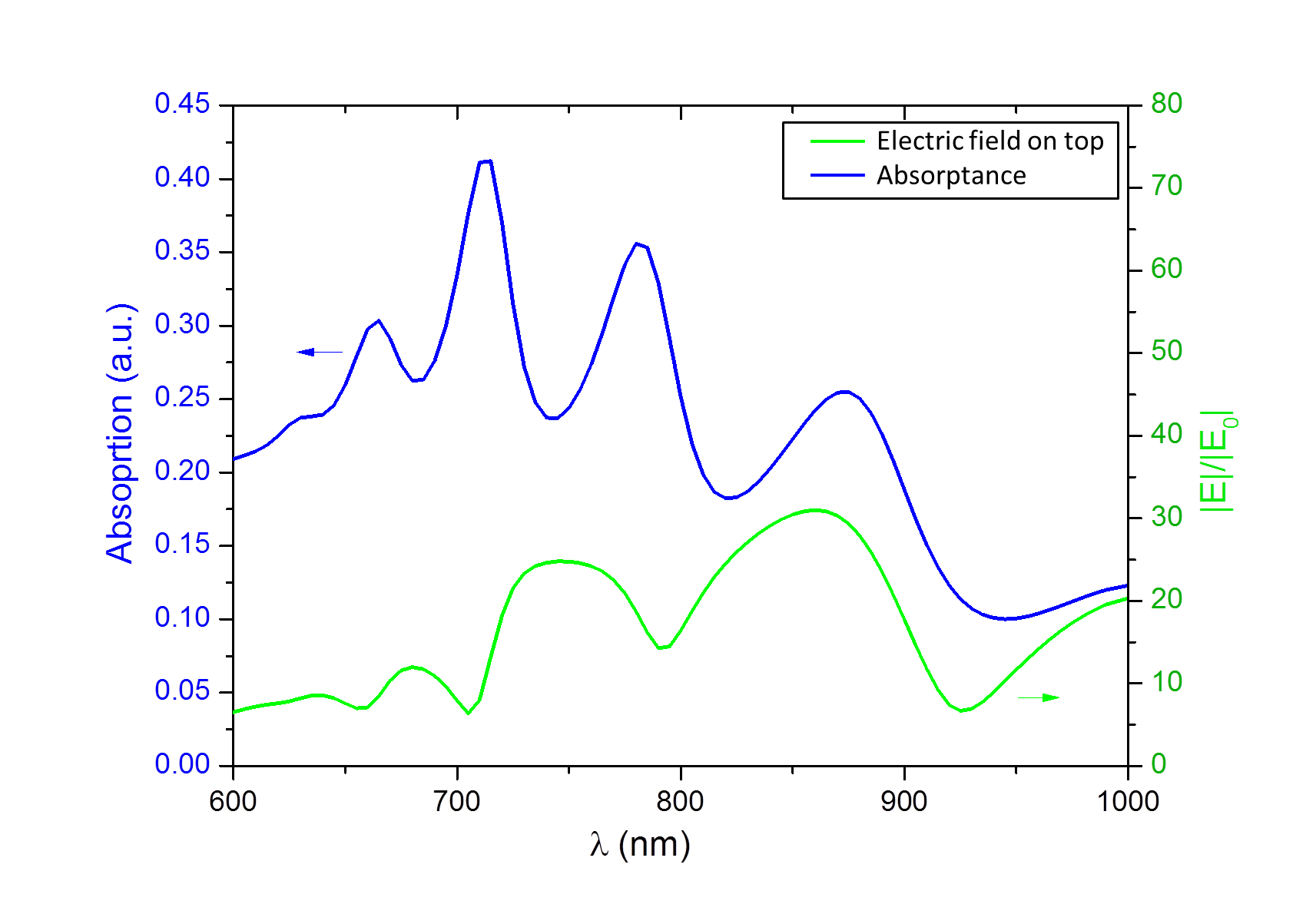
**

Figure S3: Simulated absorption spectrum of Au nanoantenna in the region of interest for the performed experiments

**References**

1 Corkum PB. Plasma perspective on strong field multiphoton ionization. *Phys Rev Lett* 1993; **71**: 1994–1997.

2 Dombi P, Hörl A, Rácz P, Márton I, Trügler A *et al*. Ultrafast Strong Field Photoemission from Plasmonic Nanoparticles. *Nano Lett* 2013; **13**: 674–678.

3 Palik ED. *Handbook of Optical Constants of Solids*. 1st ed. Academic Press, 1998.

4 Paschotta R. *Field guide to laser pulse generation*. SPIE; 2008.

5 Vogel A, Noack J, Hüttman G, Paltauf G. Mechanisms of femtosecond laser nanosurgery of cells and tissues. *Appl Phys B* 2005; **81**: 1015–1047.

6 Zolotovitskii YM, Korshunov LI, Benderskii VA. Electron work function from metals in a liquid dielectric. *Bull Acad Sci USSR Div Chem Sci* 1972; **21**: 760–763.

7 Krüger M, Schenk M, Hommelhoff P, Wachter G, Lemell C *et al*. Interaction of ultrashort laser pulses with metal nanotips: A model system for strong-field phenomena. *New J Phys* 2012; **14**: 085019.

8 Nikjoo H, Uehara S, Emfietzoglou D. *Interaction of Radiation with Matter*. New York: CRC Press; 2012.

9 Matsui M, Hoshino M, Kato H, da Silva FF, Limão-Vieira P *et al*. Measuring electron-impact cross sections of water: elastic scattering and electronic excitation of the ã^3^B_1_ and Ã^1^B_1_ states. *Eur Phys J D* 2016; **70**: 77.

10 Wiklund K, Fernández-Varea JM, Lind BK. A Monte Carlo program for the analysis of low-energy electron tracks in liquid water. *Phys Med Biol* 2011; **56**: 1985–2003.

11 Dingfelder M, Hantke D, Inokuti M, Paretzke HG. Electron inelastic-scattering cross sections in liquid water. *Radiat Phys Chem* 1999; **53**: 1–18.

12 Dingfelder M, Inokuti M. The Bethe surface of liquid water. *Radiat Environ Biophys* 1999; **38**: 93–96.

13 Emfietzoglou D, Cucinotta FA, Nikjoo H. A complete dielectric response model for liquid water: a solution of the Bethe ridge problem. *Radiat Res* 2005; **164**: 202–211.

14 Emfietzoglou D, Abril I, Garcia-Molina R, Petsalakis ID, Nikjoo H *et al*. Semi-empirical dielectric descriptions of the Bethe surface of the valence bands of condensed water. *Nucl Instruments Methods Phys Res Sect B Beam Interact with Mater Atoms* 2008; **266**: 1154–1161.

15 Emfietzoglou D, Kyriakou I, Abril I, Garcia-Molina R, Nikjoo H. Inelastic scattering of low-energy electrons in liquid water computed from optical-data models of the Bethe surface. *Int J Radiat Biol* 2012; **88**: 22–28.

16 Heller JM, Hamm RN, Birkhoff RD, Painter LR. Collective oscillation in liquid water. *J Chem Phys* 1974; **60**: 3483.

17 Dingfelder M, Ritchie RH, Turner JE, Friedland W, Paretzke HG *et al*. Comparisons of calculations with PARTRAC and NOREC: transport of electrons in liquid water. *Radiat Res* 2008; **169**: 584–594.

18 Jackson JD. *Classical Electrodynamics.* 3rd ed. Wiley;1999.

19 Hansen WN. Electric Fields Produced by the Propagation of Plane Coherent Electromagnetic Radiation in a Stratified Medium. *J Opt Soc Am* 1968; **58**: 380.

20 Devroye L. *Non-Uniform Random Variate Generation*. New York: Springer; 1986.

21 Malerba M, Alabastri A, Miele E, Zilio P, Patrini M *et al*. 3D vertical nanostructures for enhanced infrared plasmonics. *Sci Rep* 2015; **5**: 16436.

22 Zilio P, Malerba M, Toma A, Proietti Zaccaria R *et al*. Hybridization in three dimensions: a novel route towards plasmonic metamolecules. *Nano Lett* 2015; 15: 5200–5207.
